# Supplementary material for: Structural basis for self-discrimination by neoantigen-specific TCRs
Source: Nat Commun. 2024 Mar 8;15:2140. doi: 10.1038/s41467-024-46367-9 (PMC10924104; doi:10.1038/s41467-024-46367-9)
Supplement: Supplementary file 1 — Supplementary Information [file 41467_2024_46367_MOESM1_ESM.docx]

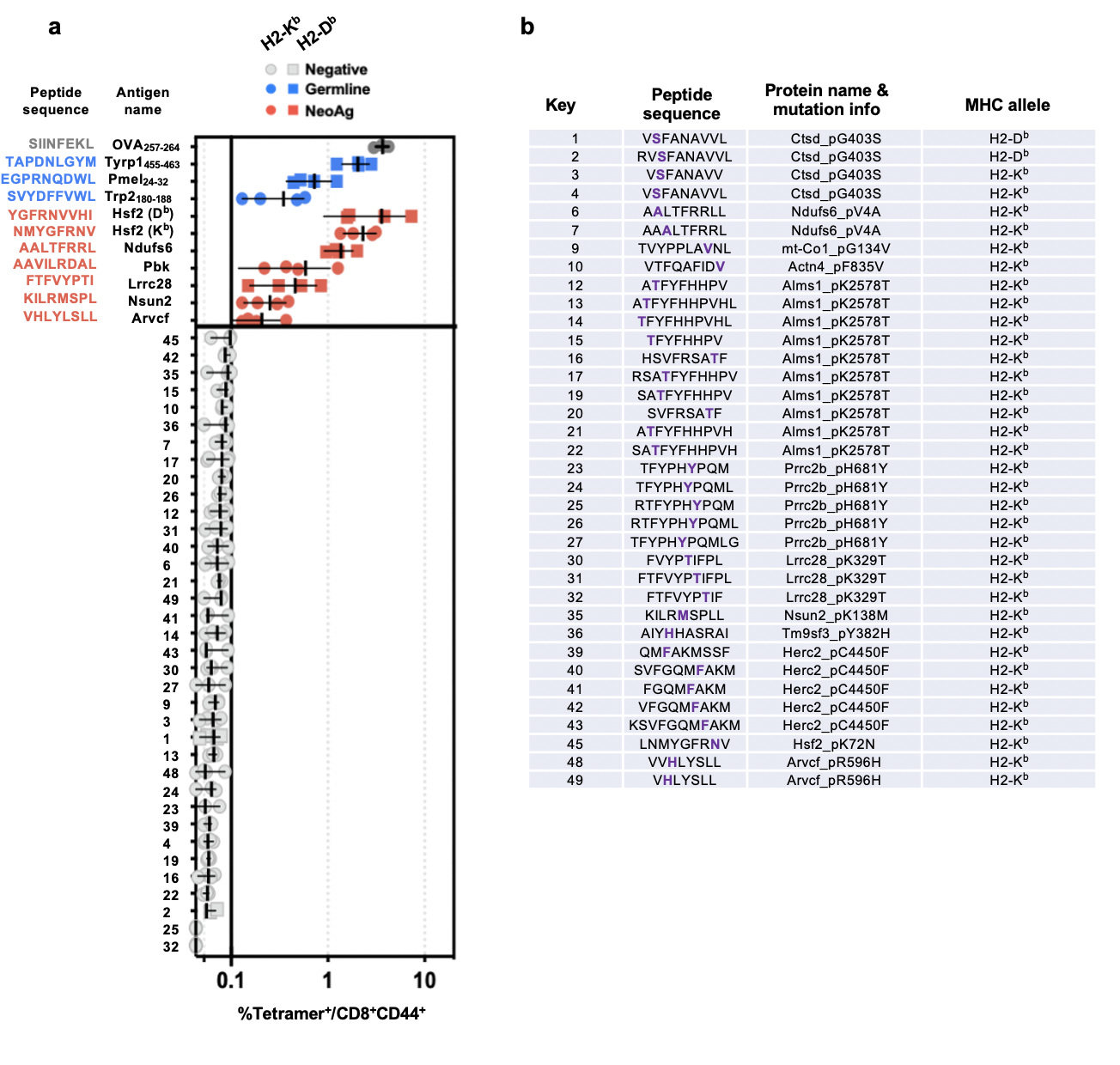


**Supplementary Fig. 1 | Identification of neoantigen-reactive T cells with peptide vaccination in non-tumour-bearing C57BL/6 mice. a**, C57BL/6 mice (n=4 independent biological replicates/group) were immunised with a single dose of peptide vaccine targeting putative B16F10 neoantigens (neoAgs) or non-mutated (germline) tumour antigens (listed, also see Fig. 1e). Putative neoAg epitopes that did not elicit a T cell response are labeled as ”negative”. Symbol indicates the frequency of pMHC tetramer-bound CD8^+^CD44^+^ T cells in peripheral blood 7 days post-immunization ± 95% confidence interval (CI). Error bars indicate the group median. **b**, Key for negative antigens shown in **a**. Bolded amino acids (purple) highlight mutated amino acid uniquely present in B16F10. Source data are provided as a Source Data file.


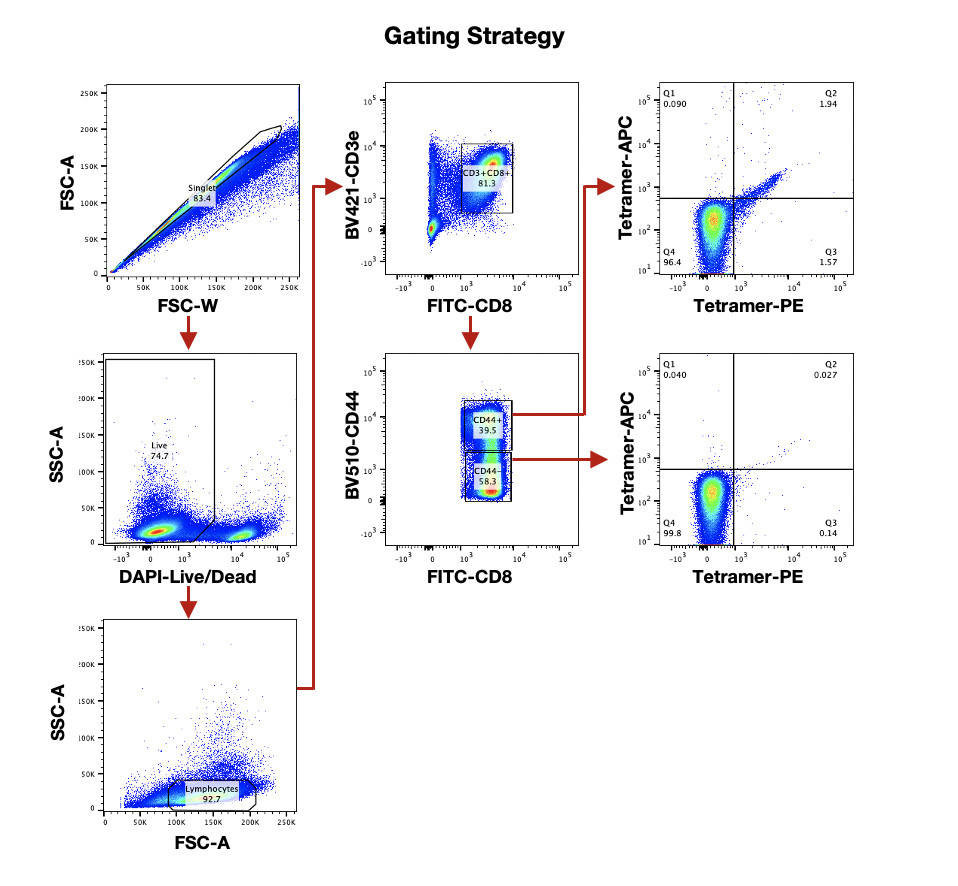


**Supplementary Fig. 2 | Flow cytometry gating corresponding to Figure 1c.** Gating strategy for flow cytometry plots showing tetramer staining of neoantigen-reactive CD8^+^ T cells isolated from vaccinated versus unvaccinated mice (Fig. 1c) is shown. Arrows indicate sequencing of gating. Tetramer staining data shown in Fig. 1c is gated on the CD44^+^ CD8^+^ population highlighted above, with the upper rightmost plot serving as a representative plot for data shown in Fig. 1c.


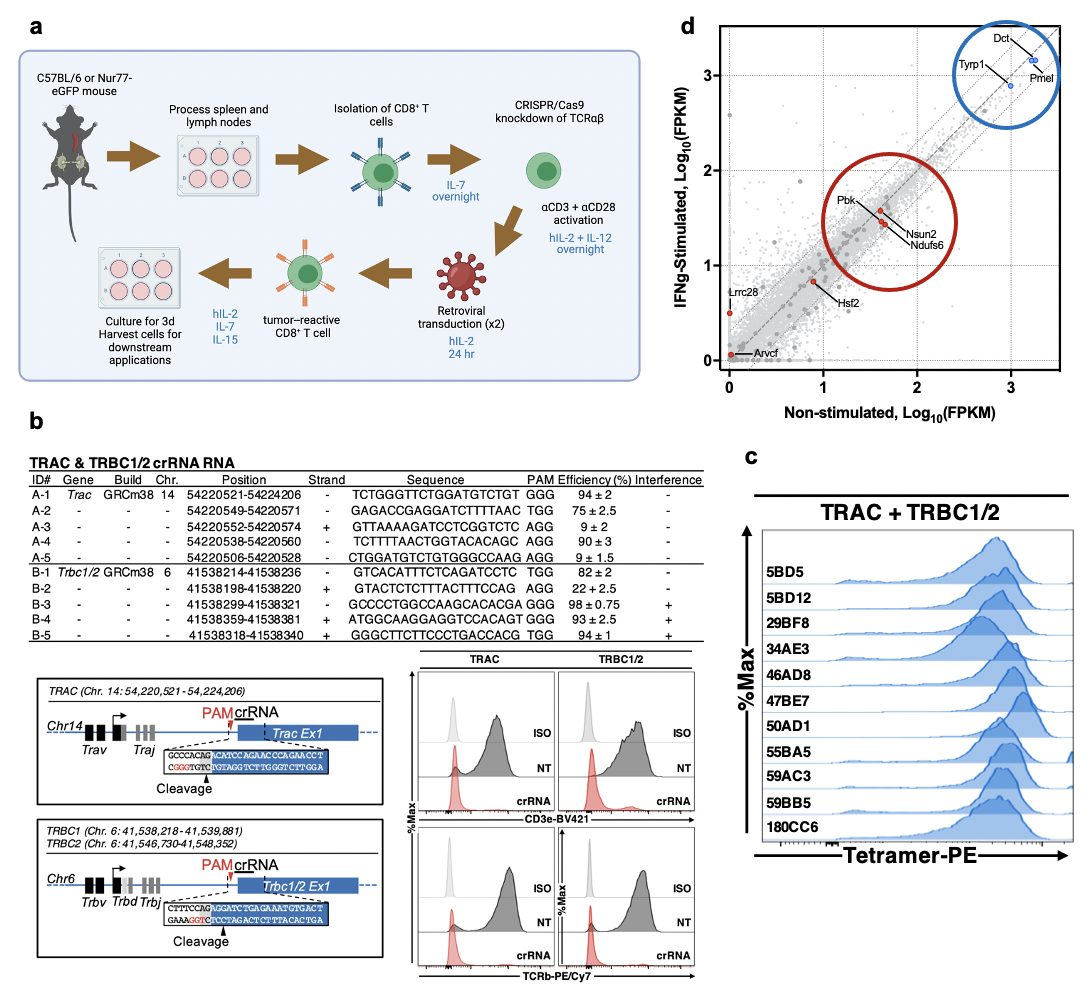


**Supplementary Fig. 3 | Design of engineered transgenic TCR (tgTCR) CD8^+^ T cells and flow cytometry data of TCR surface expression. a**, Schematic (made using Biorender with full license) depicting process of engineering transgenic TCR (tgTCR) neoantigen or tumour associated antigen-reactive (TAA) CD8^+^ T cells. hIL-2=human IL-2. **b**, Table detailing CRISPR RNA (crRNA) targeting the TRAC and TRBC loci (encoding TCR⍺ and TCRβ, respectively) (top). Diagram of cleavage sites within TRAC and TRBC is shown (bottom, left), as well as flow cytometry staining for surface TCR expression (bottom, right), comparing isotype control-stained samples (iso), cells that were not transfected with TRAC/TRBC CRISPR/Cas9 reagents (NT), and cells that underwent CRISPR/Cas9-mediated TRAC/TRBC knockdown (crRNA). **c**, Neoantigen- or TAA-reactive T cells engineered as described in **a** were analyzed by flow cytometry (gating strategy shown in Supplementary Fig. 4) after tetramer staining with neoantigen or TAA peptide-MHC monomers conjugated to phycoerythrin (PE), after dual knockdown of TRAC and TRBC loci and subsequent transduction with desired tgTCRs. TCR clone names (i.e., 5BD5, 5BD12, etc.) correspond to those listed in Fig. 2a. Data are representative of 3 independent experiments. **d**, Transcript expression of neoAg and tumour-associated antigens of relevance to our study was plotted along axes of fragments per kilobase of transcript per million mapped reads (FPKM) across unstimulated vs. IFNγ-stimulated B16F10 conditions. *Dct* is the gene encoding the protein Trp2. These data were calculated from a published dataset^70^. Source data are provided as a Source Data file.


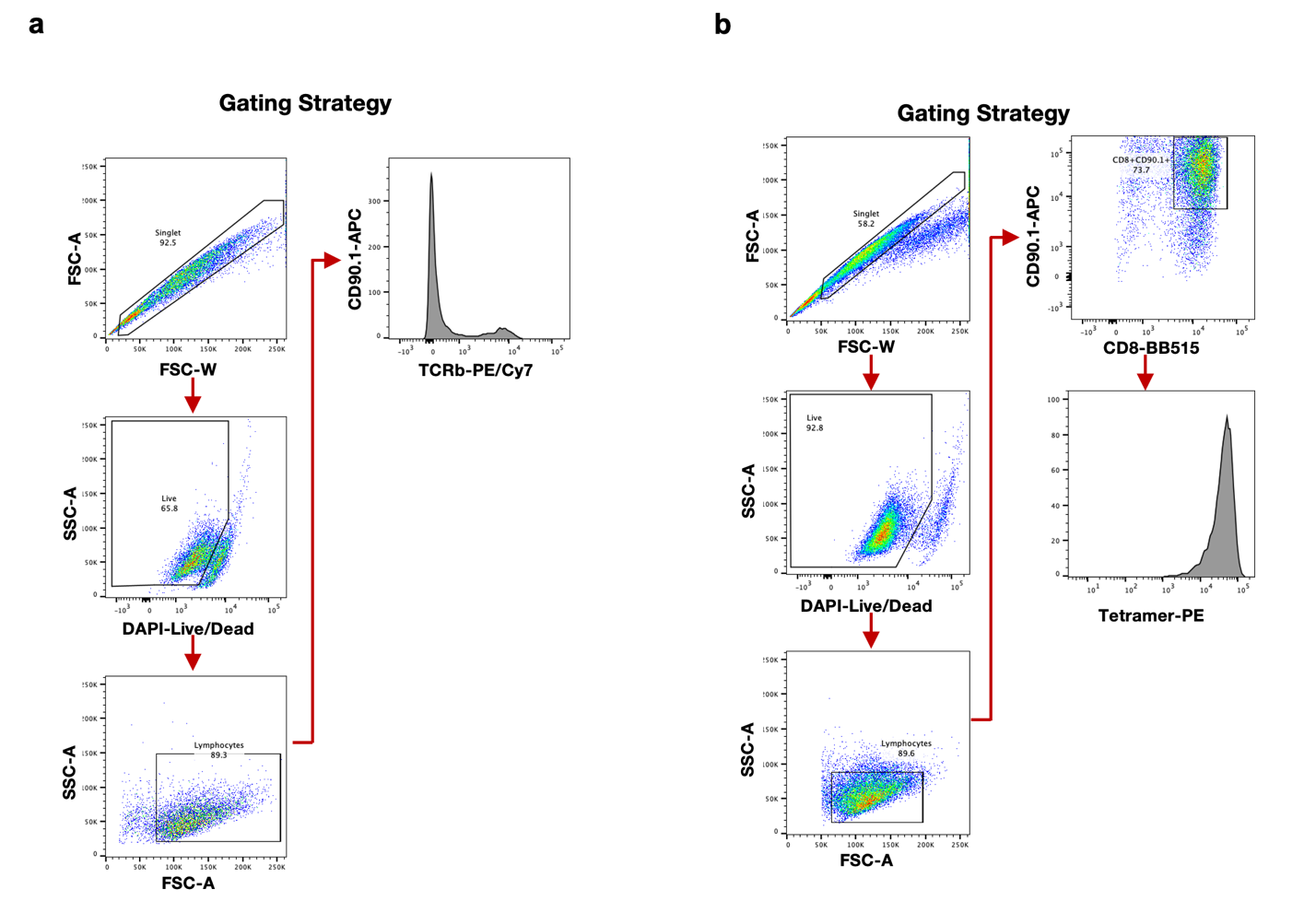


**Supplementary Fig. 4 | Flow cytometry gating strategy corresponding to Supplementary Fig. 3.** Flow cytometry gating strategies corresponding to Supplementary Fig. 3b (a) and Supplementary Fig. 3c (b) are shown. Arrows indicate sequence of gating. Final gates shown here are representative plots corresponding to the data shown in Supplementary Fig. 3b,c.


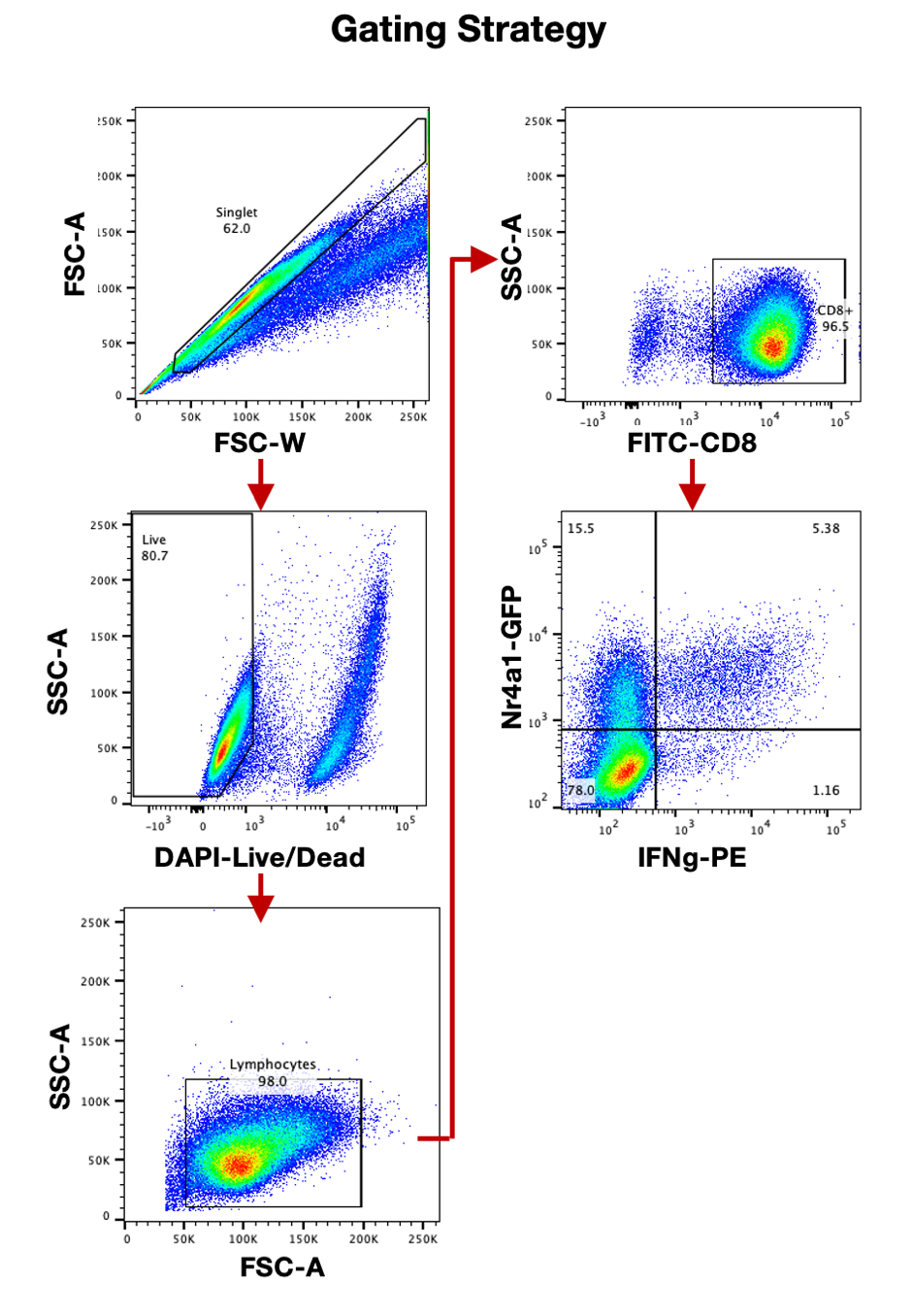


**Supplementary Fig. 5 | Flow cytometry gating strategy corresponding to Fig. 3**. Flow cytometry gating strategy is shown for data displayed in Fig. 3. Arrows indicate sequence of gating; the final plot of the gating strategy (Nr4a1-GFP vs. IFNg-PE) is representative of the flow cytometry plots shown in Fig. 3.


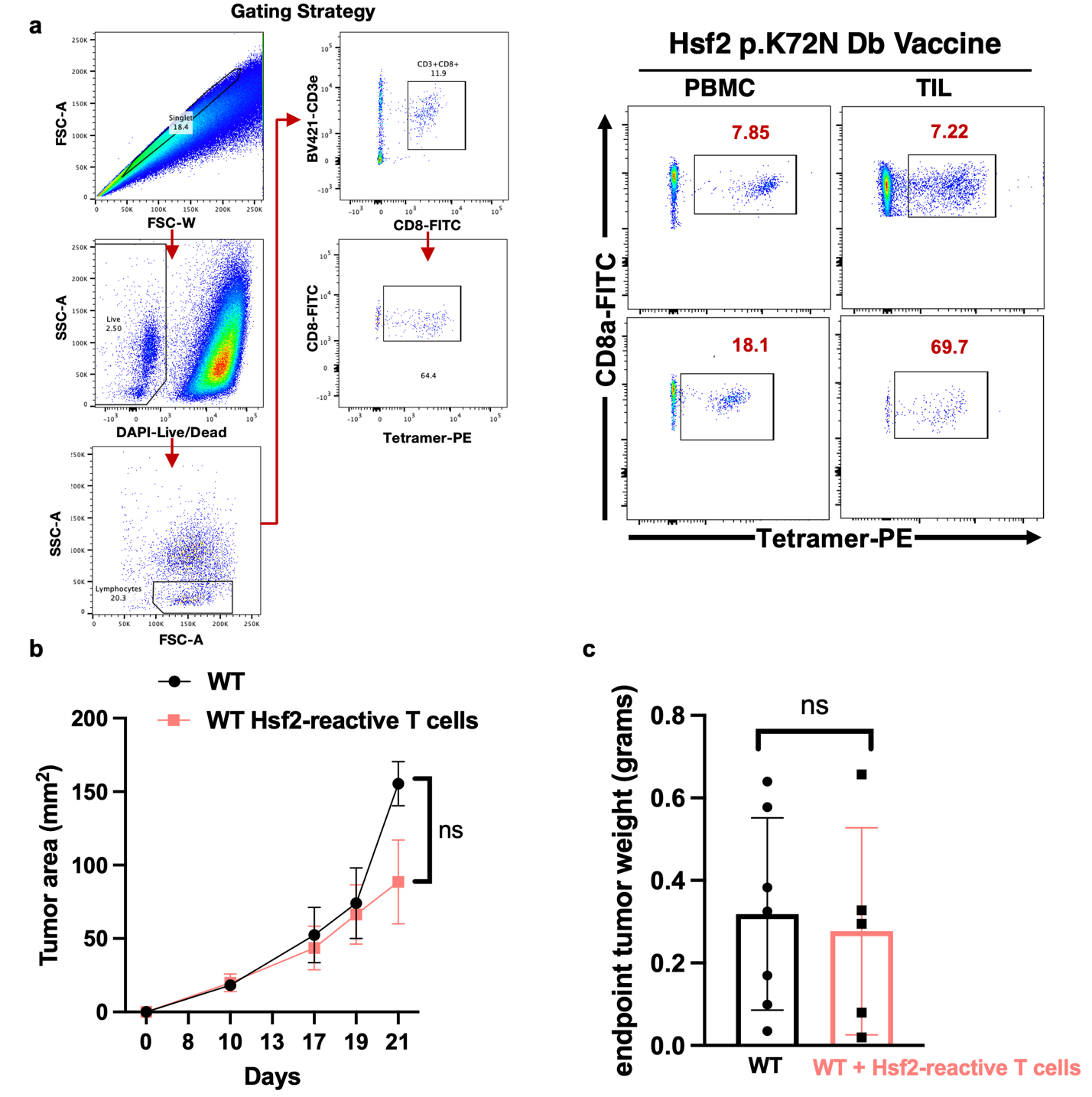


**Supplementary Fig. 6 | In vivo characteristics of vaccine-elicited immunity and adoptive cell transfer. a**, Representative flow cytometry gating strategy (left) and plots (right) of tetramer staining of T cells from peripheral blood mononuclear cells (PBMCs) or tumour-infiltrating lymphocytes (TILs) corresponding to Fig. 4b. Each row represents a distinct Hsf2-vaccinated mouse analyzed. Data are representative of two independent experiments. **b**, C57BL/6 mice (n=5 [WT+Hsf2-reactive T cells] or 6 [WT group] independent biological replicates) received 2.0×10^5^ wild type (WT) B16F10 cells (harbouring natively low expression of Hsf2 neoantigenic epitope) intradermally in the flank, were irradiated (whole body, 5 Gy) 6 days later, and administered 2.0×10^7^ 47BE7 Hsf2-reactive CD8^+^ T cells (ACT) or phosphate-buffered saline (Vehicle) intravenously (i.v.) one day later. hIL-2 was administered at 180,000 IU/mouse on day of ACT and daily for 2 subsequent days; supplementary doses were administered weekly until humane endpoint. **c**, Mice (n=5 [WT+Hsf2-reactive T cells] or 7 [WT] independent biological replicates/group) were sacrificed at humane endpoint and tumours were weighed, as shown. Ns= not significant, two-way ANOVA with Bonferroni correction, *p*=0.4145 (**b**) or student’s t-test (two-tailed), *p*=0.7685 (**c**). Error bars depict standard error of the mean.


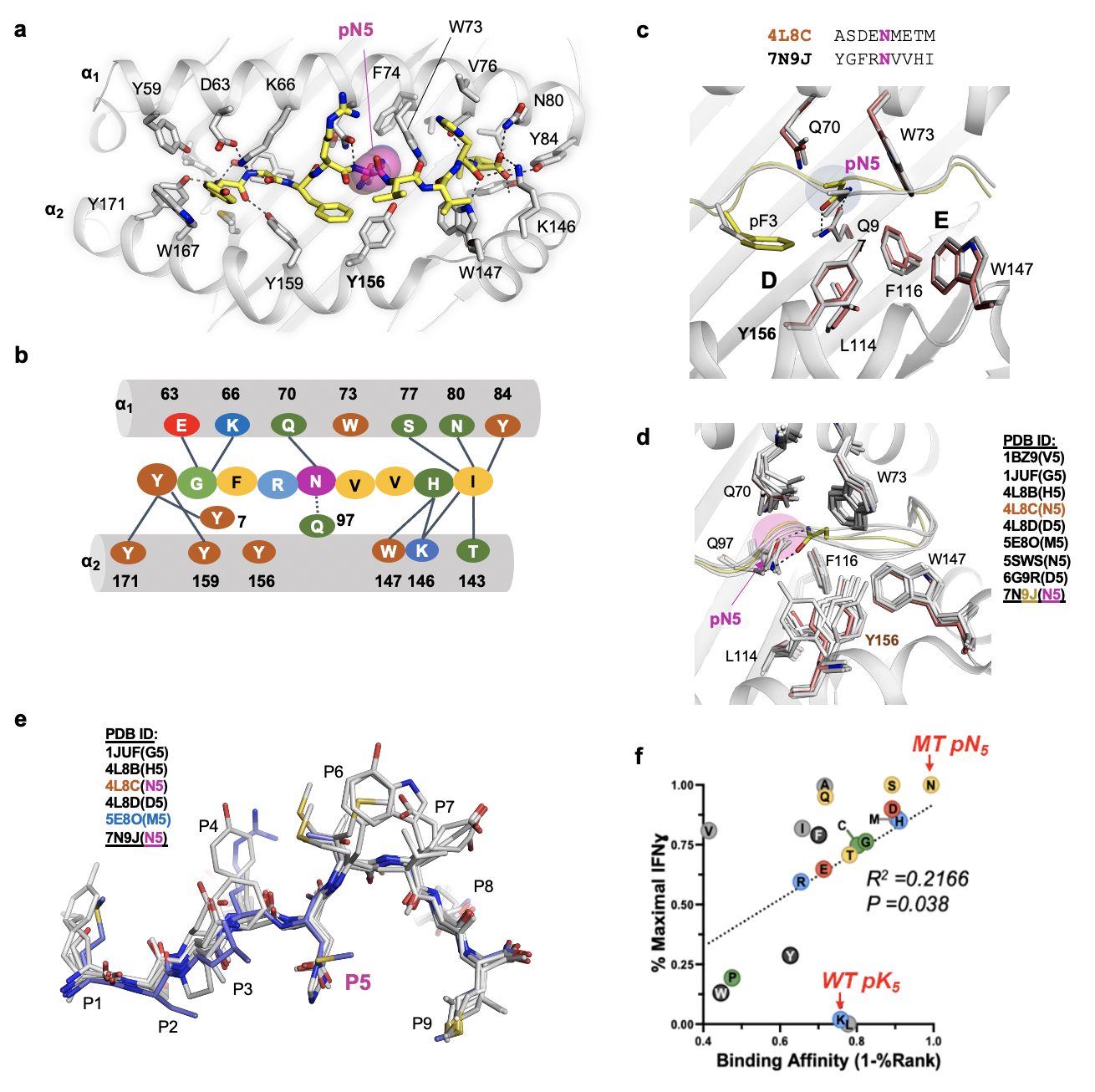


**Supplementary Fig. 7**. **Binding between peptides and H2-D^b^**. **a**, The polar interaction network between bound epitope and H2-D^b^. Amino acid residues are presented as sticks and hydrogen bonds (distance cutoff < 3.5 Å) are shown with dotted lines. **b**, The polar interaction network, a colored scheme. Residues are color-coded based on molecular properties; red (acidic), blue (basic), brown (aromatic), green (polar), yellow (nonpolar). **c**, Structural superimposition of PDB 7N9J [[https://www.rcsb.org/structure/7n9j])] (carbons are grey and in peptide – yellow) and 4L8C (cartoon and stick models), C pocket of H2-D^b^. **d**, Superimposition between atomic coordinates of the H2-D^b^ crystal structures from the PDB in complex with various epitopes, cartoon and sticks, Hsf2 p.K72N_68-72_ carbon atoms are shown in yellow. The PDB files are listed in the figure; amino acid residues in p5 are shown in parentheses. 7N9J [https://www.rcsb.org/structure/7n9j] is the structure of H2-D^b^ in complex with Hsf2 p.K72N_68-72_ epitope described here. **e**, Superimposition between atomic coordinates of the various peptides complexed with H2-D^b^, stick representation. The PDB files are listed in the figure, the amino acid residue at p5 is shown in parentheses. **f**, Dot plot of position 5 peptide variants from the positional scanning library plotted according to in silico MHC-I binding affinity and measured %IFNγ^+^TCR-47BE7^+^CD8^+^ T cells by intracellular cytokine staining (Fig. 5f). Colored according to side chain biochemical property. Polar uncharged (yellow), Positive charge/acidic (red), negative charge/basic (blue), hydrophobic (grey), aromatic (black), special (green). MT= mutant, WT= wild type. Source data are provided as a Source Data file.


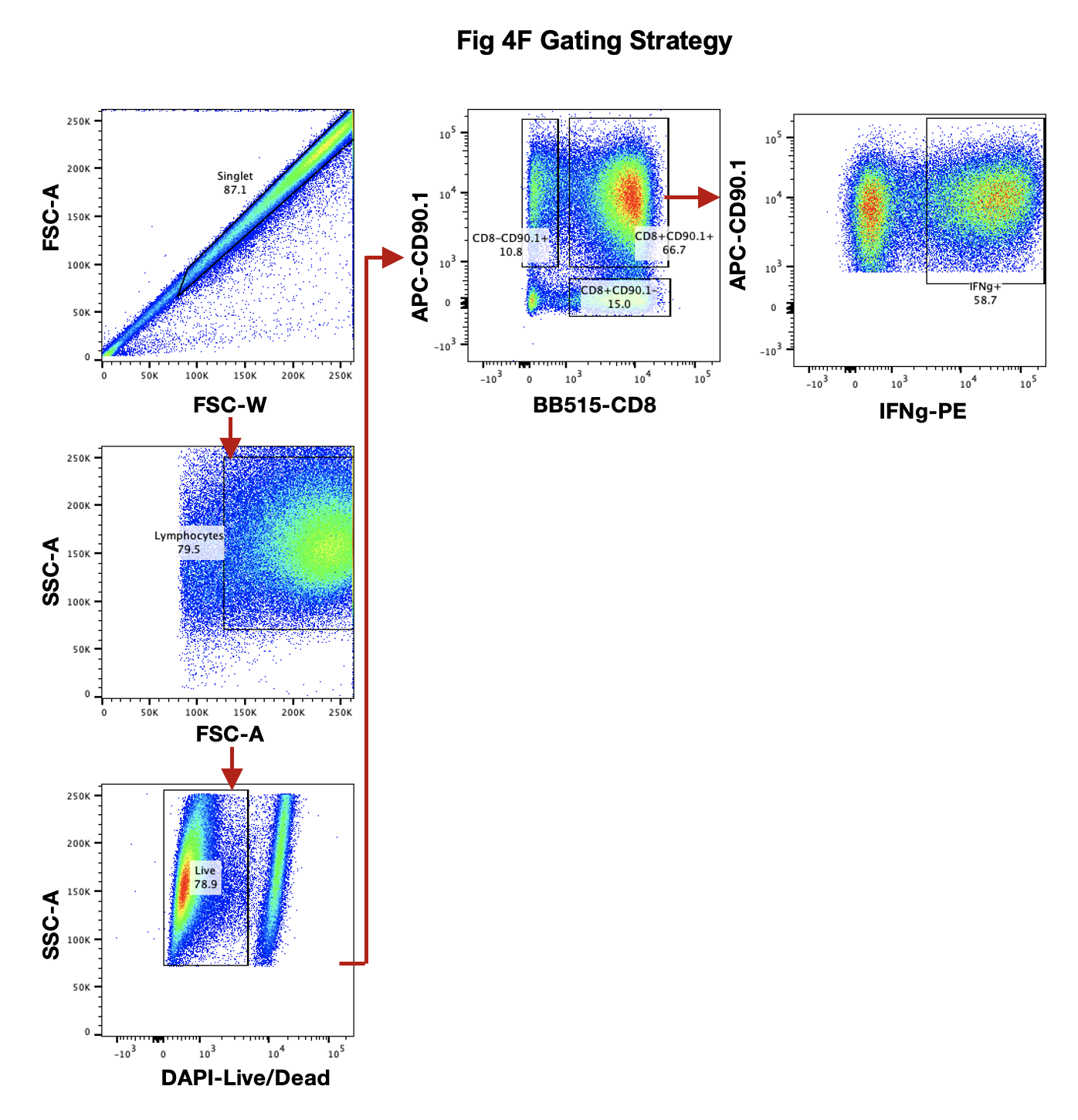


**Supplementary Fig. 8. Flow cytometry gating corresponding to Fig. 5f.** Flow cytometry gating corresponding to data plotted in Fig. 5f; all data shown in Fig. 5f are calculated from the IFNg^+^ CD90.1^+^ population after CD90.1^+^CD8^+^ gating (see right for representative flow cytometry plot). CD90.1 expression is a marker for Hsf2-reactive T cells, as CD90.1 and Hsf2-reactive TCRs are dually encoded by the retrovirus used to transduce CD8^+^ T cells (see methods).


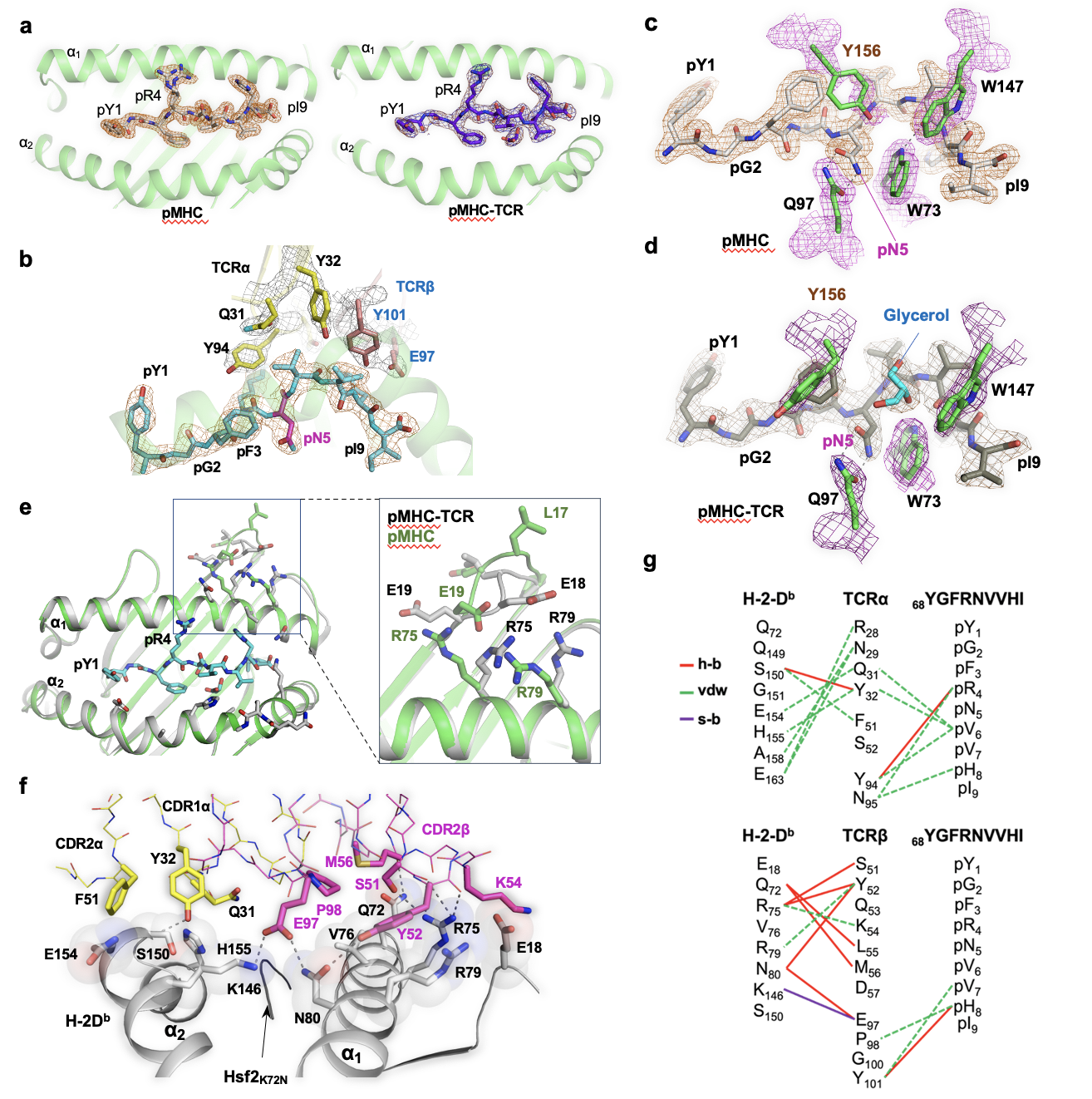


**Supplementary Fig. 9**. **pMHC-TCR interface.** Nitrogens are colored in blue and oxygens in red. **a**, The SigmaA-weighted Fo-Fc maps (σ=4.0, radius=1.5Å) around peptides bound to H2-D^b^ in the pMHC or pMHC-TCR 47BE7, respectively. Cartoon and stick models. **b**, The SigmaA-weighted 2Fo-Fc map (σ=1.0, radius=1.6Å) around selected amino acid residues at the pMHC-TCR 47BE7 interface. The epitope carbon atoms are blue (pN_5_ is magenta), TCRα - yellow, TCRβ - orange. **c** and **d**, The SigmaA-weighted 2Fo-Fc maps (σ=1.0, radius=1.6Å) around selected residues in pMHC and pMHC-TCR 47BE7 structures, respectively. Stick models. The maps around the H2D^b^ residues are colored in magenta, and the carbon atoms are colored in green. **e**, Superposition of H2-D^b^ atomic coordinates from the binary and ternary complexes, and H2-D^b^ loop A (inlet). **d**, Interface between TCR-47BE7 and H2-D^b^ in the ternary complex. Amino acid residues located at the TCR-MHC interface (distance cutoff <4 Å) are displayed as sticks, interacting H2-D^b^ residues are shown as semi-transparent spheres, and other residues are presented as wires. Hydrogen bonds are depicted as dotted lines. **g**, Interaction pattern at the pMHC-TCR 47BE7 interface. h-b – hydrogen bonds, VDW– Van-der-Waals bonds, s-b – salt bridge.

**
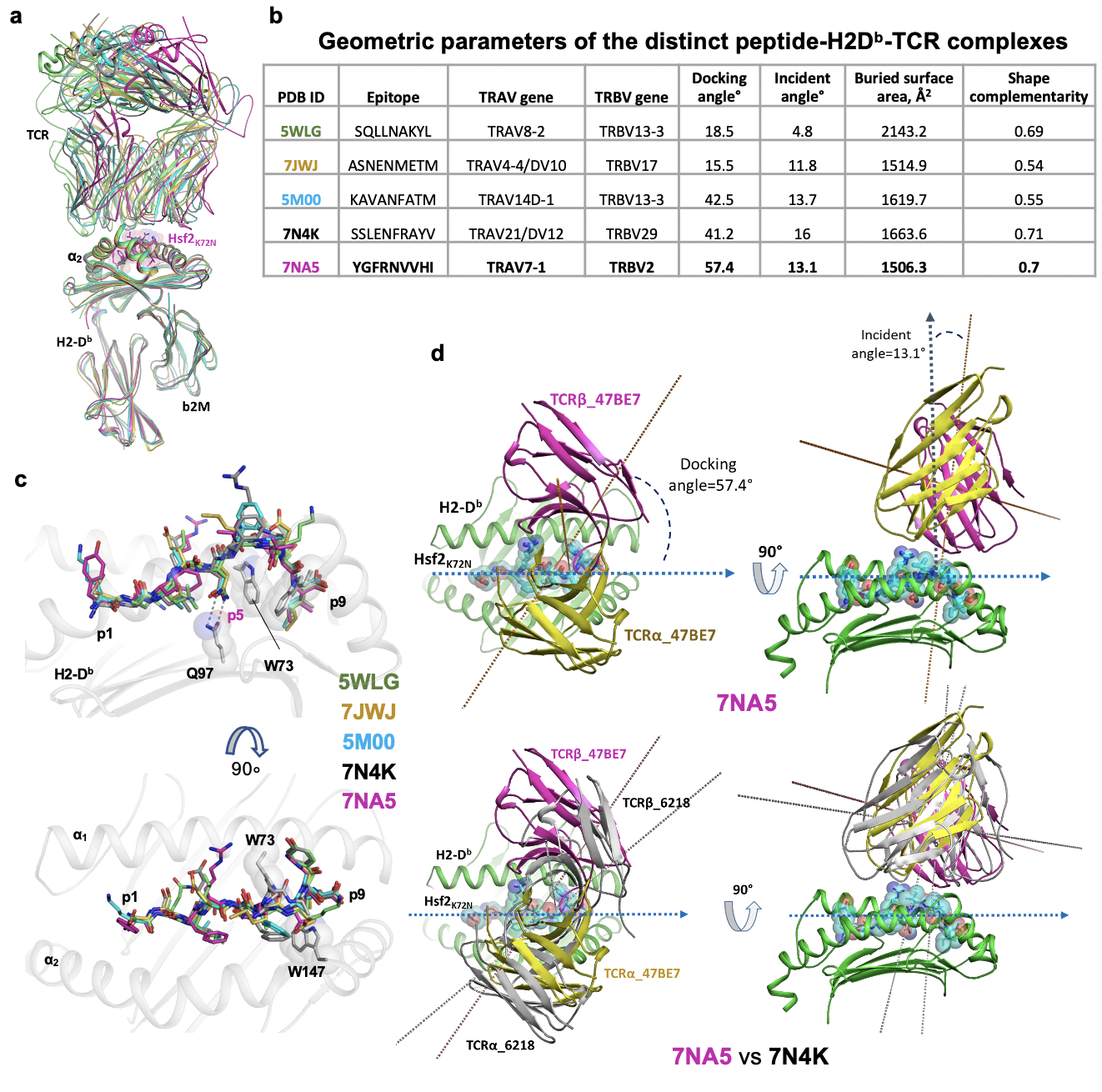
**

**Supplementary Fig. 10. Relative orientation of TCR with respect to pMHC is dependent on the nature of epitope in the ternary complex. a,** Structural superimposition of the distinct pMHC-TCR structures listed in (**b**). Cartoon models. Each structure is colored according to the PDB ID. Superposition was performed using coordinates of the H2-D^b^ molecules. **b**, Geometric parameters for the distinct peptide-H2D^b^-TCR complexes calculated using NACCESS as described in Methods. **c**, Peptide ligands from the distinct peptide-H2D^b^-TCR complexes superposed as shown in (**a**), the two orientations are related by the ~90° rotation around the MHC groove vector. Carbon atoms are colored according to PDB IDs. **d**, Docking and incident angles are shown for the two least dissimilar structures, 7NA5 [https://www.rcsb.org/structure/7NA5] and 7N4K [https://www.rcsb.org/structure/7N4K], respectively. Calculations were performed as described in the following cited publication^1^.

**
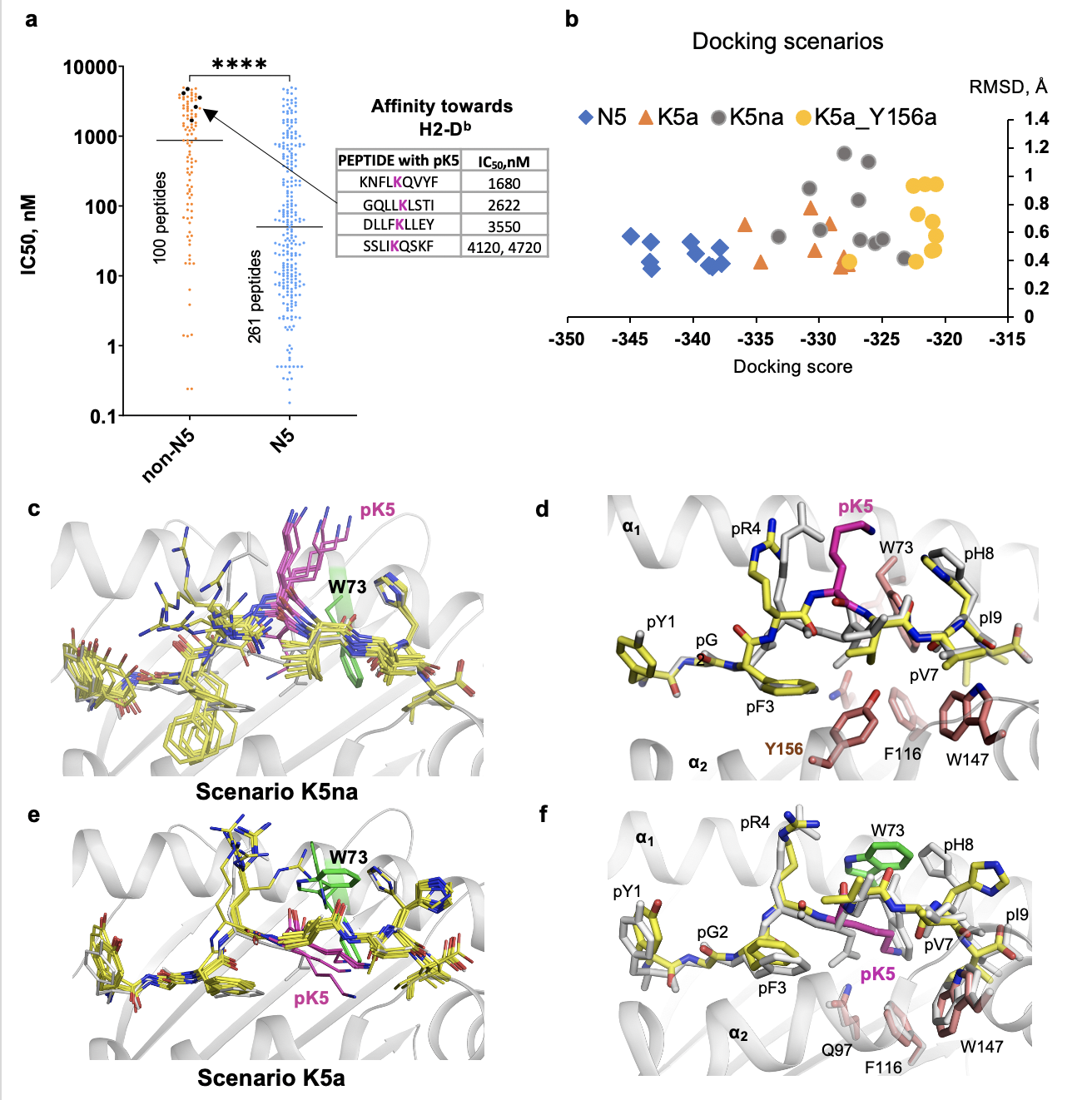
**

**Supplementary Fig. 11. Binding of pK5-peptides to H2-D^b^.** Cartoon representation. Peptides are shown as sticks. Peptide carbon atoms (except for pLys_5_) are yellow, the H2-D^b^ side chain carbon atoms are colored in orange, nitrogen atoms are blue, oxygen atoms are red. H2-D^b^ Trp_73_ carbons are colored green. **a**, Affinity between H2-D^b^ and different 9AA residue peptides, split into the two groups, pN_5_ or non-pN_5_, respectively. The data were retrieved from the IEDB (<https://www.iedb.org/>) and processed using Prism. The table depicts 9AA peptides with pK5, and the graph show them as black dots. The lines represent the median values. The unpaired T-test was used to determine the difference between the two sets in Prism. **** - *p*<0.0001. **b**, Docking scores for 10 top solutions in each docking scenario. **c**, Superimposition of the top 10 solutions for pKna docking scenario. **d**, The top solution for the pKna scenario. **e**, Superimposition of the top 10 solutions for pKa docking scenario. **f**, The top solution for the pKa scenario.

**
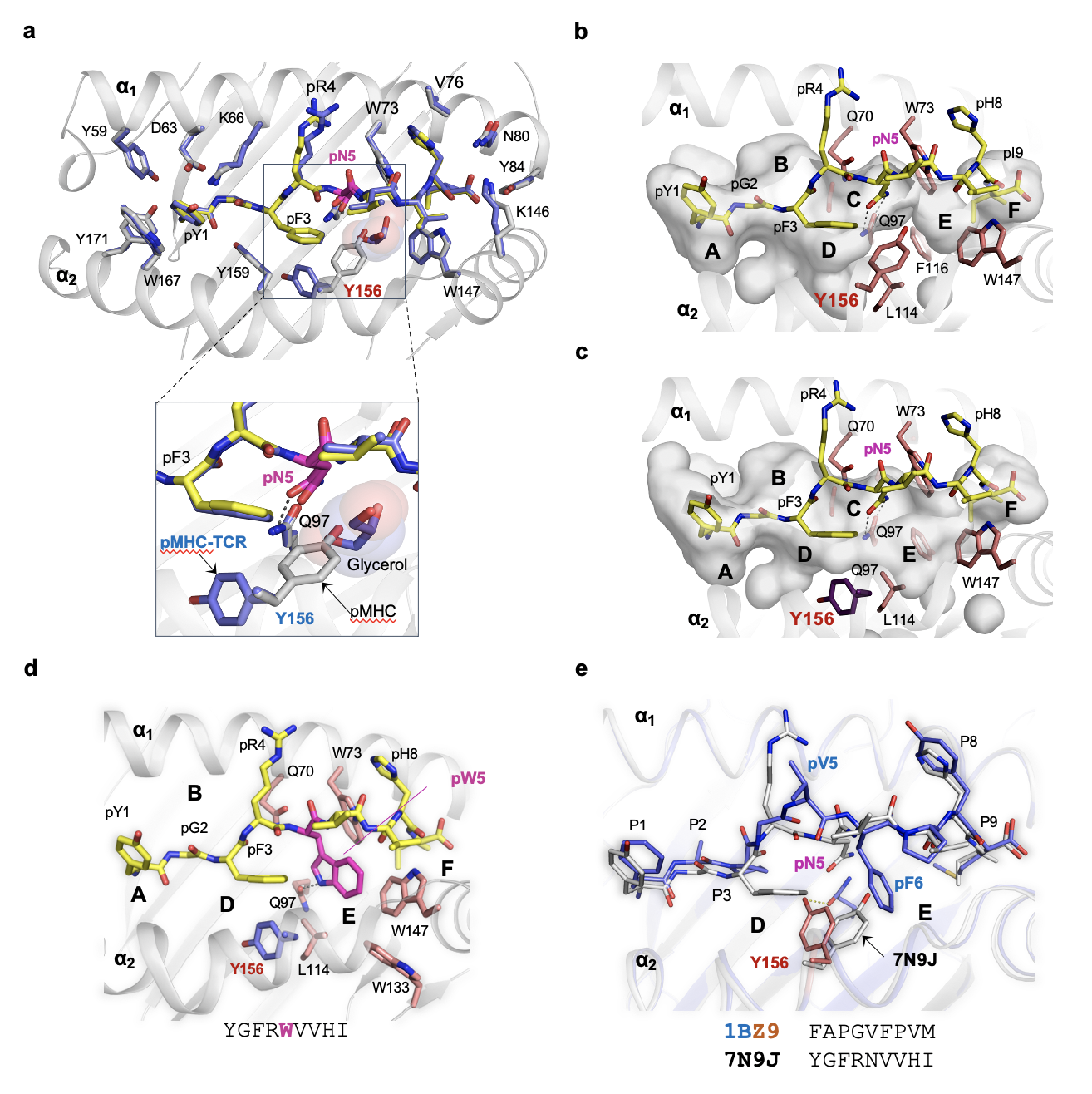
**

**Supplementary Fig. 12. Flexibility of H2-D^b^ Y_156_ may allow for alternate peptide binding mode**. Cartoon and stick representation. Peptide carbon atoms are yellow (structure 7N9J, [https://www.rcsb.org/structure/7n9j] binary complex) or light blue (structure 7NA5 [https://www.rcsb.org/structure/7NA5], pMHC-TCR), nitrogen atoms are dark blue, oxygen atoms are red, pN5 residue shown in pink. **a**, Structural superposition of pMHC (peptide carbons are yellow, H2-D^b^ carbons are grey) and pMHC-TCR (H2-D^b^ carbons are light blue). Bound glycerol is presented as sticks and semi-transparent spheres. **b** and **c**, The shape of peptide binding cavity (semi-transparent surface) in H2-D^b^ is dependent on Y_156_ conformation (glycerol was omitted). **d**, The model between H2-Db and the Hsf2_68-76_ p.K72W peptide was produced by PepFlexDock using coordinates of the structure 7N9J [https://www.rcsb.org/structure/7n9j]. Cartoon and stick model. **e**, Structural alignment of H2-Db in complex with Hsf2_68-76_ p.K72N (7N9J [https://www.rcsb.org/structure/7n9j], carbon atoms are grey colored) and synthetic peptide FAPGVFPVM (1BZ9).

**Supplementary Table 1**

**List of long peptide sequences utilised for peptide vaccination studies in Fig. 1**

**
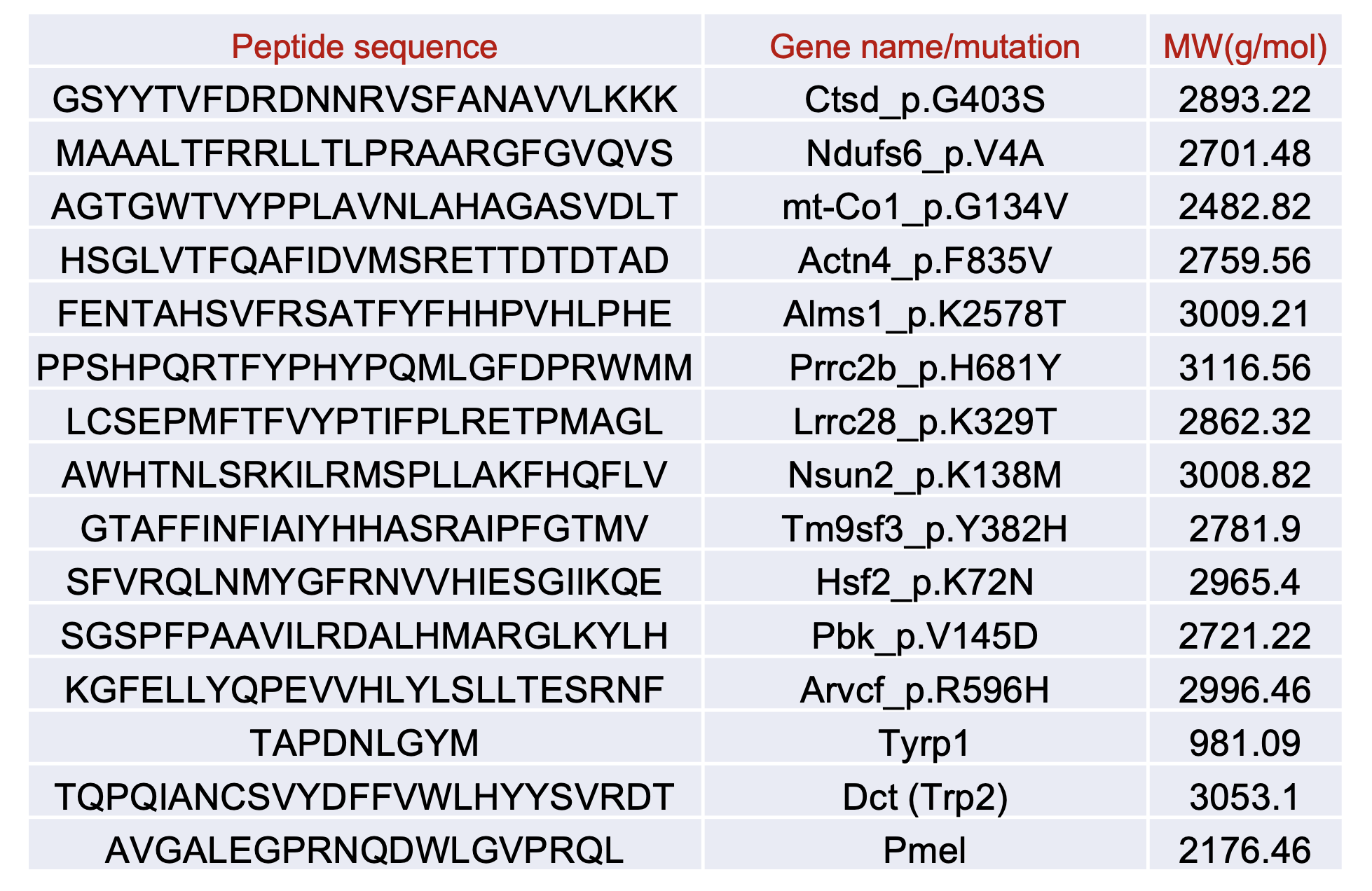
**

**Supplementary Table 2**

**X-ray Data Collection and Refinement Statistics**

**
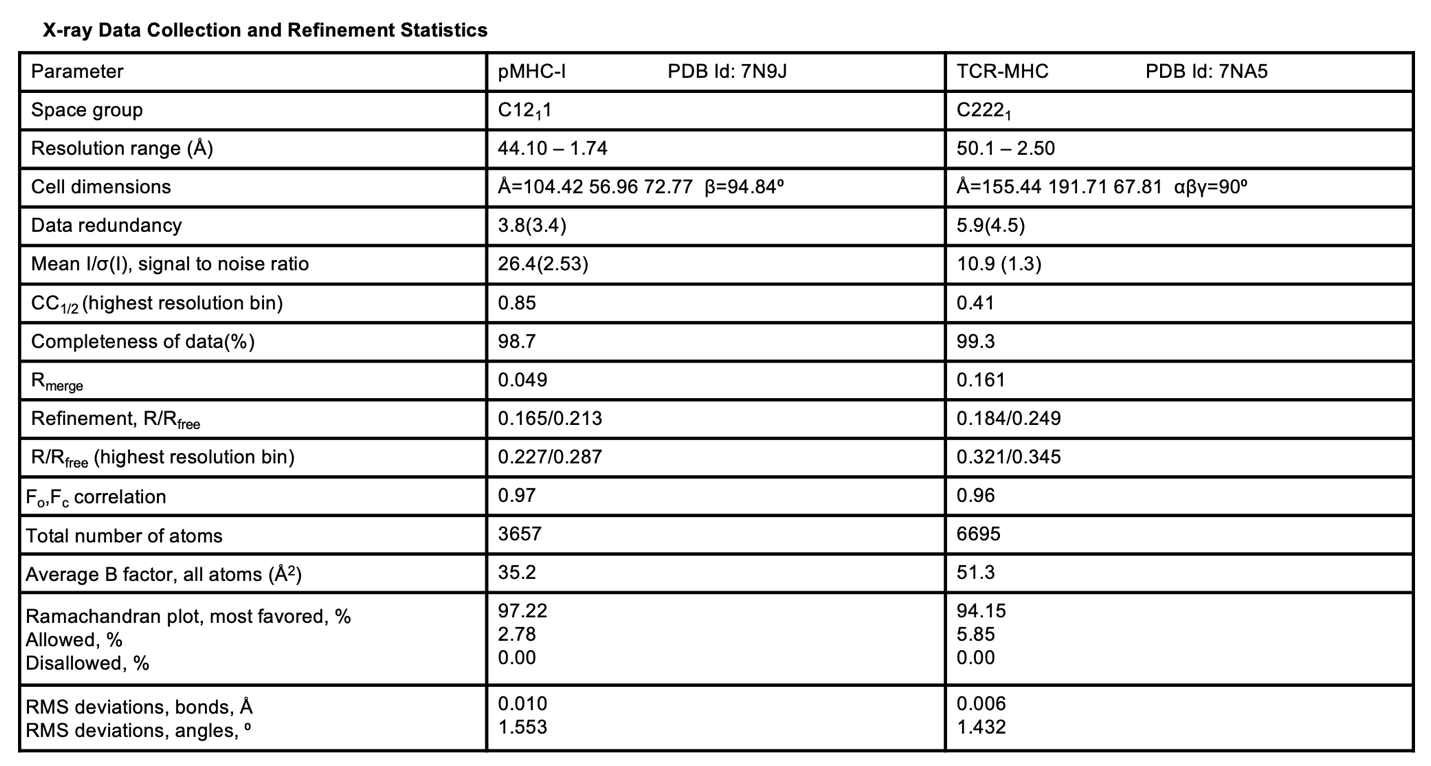
**

**Supplementary Table 3**

**cDNA sequences encoding long peptides in lentiviral constructs used to transduce B16F10 to create antigen-overexpressing B16F10**

**
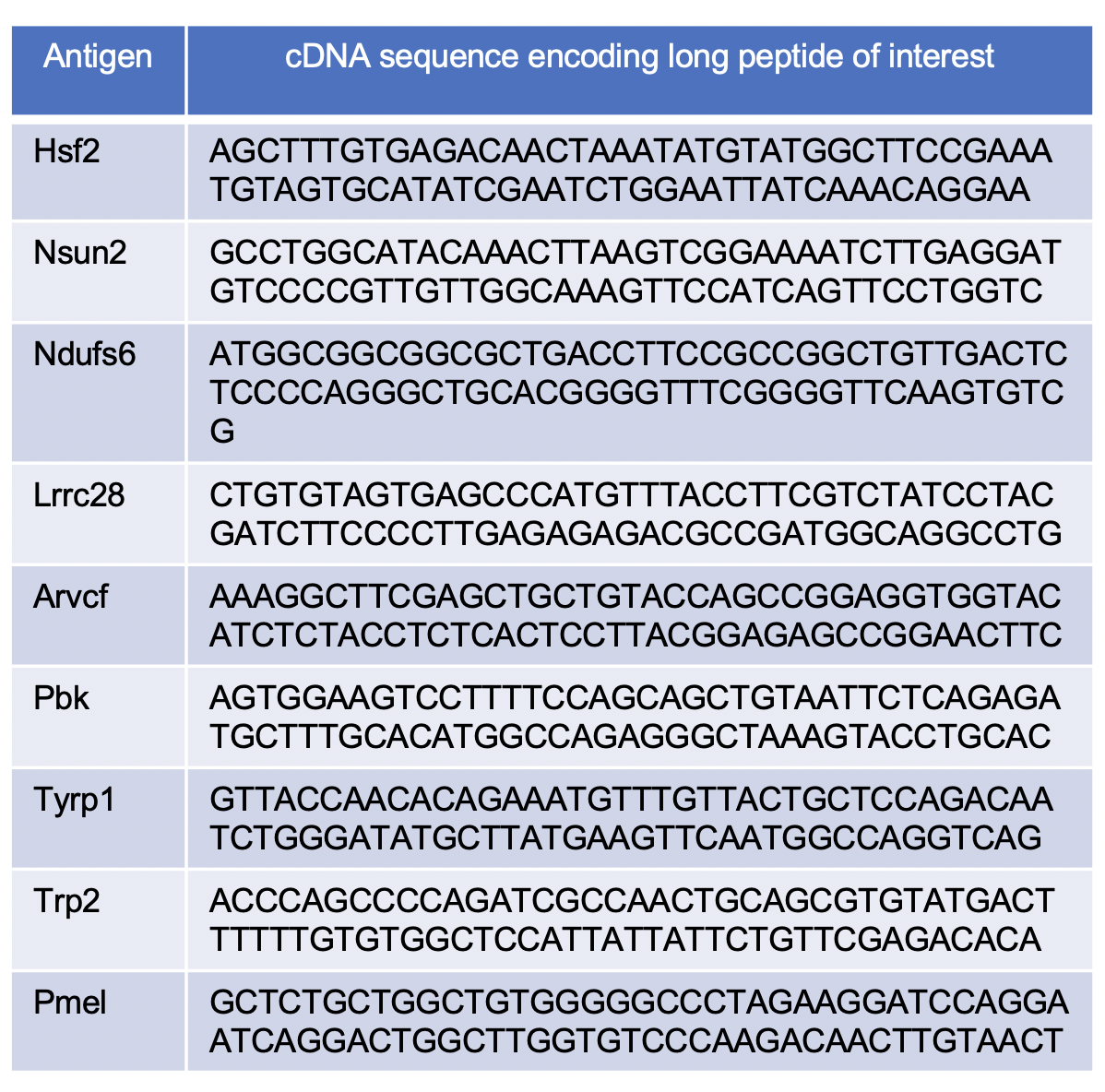
**
