# Supplementary material for: Structural basis for self-discrimination by neoantigen-specific TCRs
Source: Nat Commun. 2024 Mar 8;15:2140. doi: 10.1038/s41467-024-46367-9 (PMC10924104; doi:10.1038/s41467-024-46367-9)
Supplement: Supplementary file 3 — Description of Additional Supplementary files [file 41467_2024_46367_MOESM3_ESM.pdf]

Description of Additional Supplementary Files

File Name: Supplementary Data 1
ProSite Pattern Search Results
