# Supplementary material for: Structural basis for self-discrimination by neoantigen-specific TCRs
Source: Nat Commun. 2024 Mar 8;15:2140. doi: 10.1038/s41467-024-46367-9 (PMC10924104; doi:10.1038/s41467-024-46367-9)
Supplement: Supplementary file 4 — Supplementary Dataset 1 [file 41467_2024_46367_MOESM4_ESM.pdf]

Pos	Peptide	ID	H-2-Db core	icore	EL-score	EL_Rank	BA-score	BA_Rank
0	NVFRNILHV	PEPLIST	NVFRNILH	NVFRNILH	0.4526	0.1322	0.4209	0.1945
0	NVFRNILHV	PEPLIST	NVFRNILH	NVFRNILH	0.4526	0.1322	0.4209	0.1945
0	NVFRNILHV	PEPLIST	NVFRNILH	NVFRNILH	0.4526	0.1322	0.4209	0.1945
0	NVFRNILHV	PEPLIST	NVFRNILH	NVFRNILH	0.4526	0.1322	0.4209	0.1945
0	RAWRDILAL	PEPLIST	RAWRDILA	RAWRDILA	0.2998	0.2822	0.3162	0.5021
0	AGYRSIMHL	PEPLIST	AGYRSIMH	AGYRSIMH	0.2652	0.333	0.2511	0.9767
0	AGYRSIMHL	PEPLIST	AGYRSIMH	AGYRSIMH	0.2652	0.333	0.2511	0.9767
0	YMCRIIVHF	PEPLIST	YMCRIIVH	YMCRIIVH	0.0435	2.3523	0.1332	4.1621
0	GSWRDVLAV	PEPLIST	GSWRDVL	GSWRDVL	0.0284	3.3335	0.1331	4.1643
0	LSRRHILHL	PEPLIST	LSRRHILHL	LSRRHILHL	0.0319	3.0246	0.1194	5.2209
0	SIFRAVLHF	PEPLIST	SIFRAVLHF	SIFRAVLHF	0.0384	2.6076	0.119	5.2488
0	ILYRAVMAI	PEPLIST	ILYRAVMA	ILYRAVMA	0.0093	7.4455	0.1184	5.305
0	ILYRAVMAI	PEPLIST	ILYRAVMA	ILYRAVMA	0.0093	7.4455	0.1184	5.305
0	TFFRQVLAL	PEPLIST	TFFRQVLA	TFFRQVLA	0.0244	3.749	0.1146	5.6634
0	RTKRDILAL	PEPLIST	RTKRDILAL	RTKRDILAL	0.0445	2.309	0.1019	7.0563
0	RTKRDILAL	PEPLIST	RTKRDILAL	RTKRDILAL	0.0445	2.309	0.1019	7.0563
0	GGRRHVVHM	PEPLIST	GGRRHVV	GGRRHVV	0.0263	3.5196	0.1012	7.1524
0	EIKRNIMAL	PEPLIST	EIKRNIMA	EIKRNIMA	0.0404	2.4885	0.1005	7.2549
0	AFYRAVLAL	PEPLIST	AFYRAVLA	AFYRAVLA	0.0174	4.8738	0.0955	7.9783
0	GSYRCVVHC	PEPLIST	GSYRCVVH	GSYRCVVH	0.01	7.1321	0.0946	8.1148
0	GSYRCVVHC	PEPLIST	GSYRCVVH	GSYRCVVH	0.01	7.1321	0.0946	8.1148
0	LLRRDVVHI	PEPLIST	LLRRDVVH	LLRRDVVH	0.0078	8.3608	0.0866	9.5573
0	LLRRDVVHI	PEPLIST	LLRRDVVH	LLRRDVVH	0.0078	8.3608	0.0866	9.5573
0	ATFRCILAT	PEPLIST	ATFRCILAT	ATFRCILAT	0.0028	15.379	0.0849	9.8745
0	LARRHVLAA	PEPLIST	LARRHVLA	LARRHVLA	0.0048	11.3012	0.0845	9.9519
0	LARRHVLAA	PEPLIST	LARRHVLA	LARRHVLA	0.0048	11.3012	0.0845	9.9519
0	EARRHILAI	PEPLIST	EARRHILAI	EARRHILAI	0.012	6.3137	0.0833	10.2062
0	LSRRFILHV	PEPLIST	LSRRFILHV	LSRRFILHV	0.0064	9.5029	0.0826	10.3528
0	SIRRQILAL	PEPLIST	SIRRQILAL	SIRRQILAL	0.0142	5.6215	0.0808	10.7562
0	LPCRHILAL	PEPLIST	LPCRHILAL	LPCRHILAL	0.0105	6.908	0.0685	14.1037
0	LPCRHILAM	PEPLIST	LPCRHILA	LPCRHILA	0.0083	8.0142	0.0672	14.5884
0	CACRDVLAW	PEPLIST	CACRDVLA	CACRDVLA	0.0047	11.3713	0.0616	16.6353
0	EGRRIIVAI	PEPLIST	EGRRIIVAI	EGRRIIVAI	0.0065	9.4107	0.0575	18.4758
0	AFFRAVVAF	PEPLIST	AFFRAVVA	AFFRAVVA	0.0058	10.1453	0.057	18.6616
0	AFFRAVVAF	PEPLIST	AFFRAVVA	AFFRAVVA	0.0058	10.1453	0.057	18.6616
0	DIFRIIMAI	PEPLIST	DIFRIIMAI	DIFRIIMAI	0.0031	14.4191	0.0542	20.0842
0	DIFRIIMAI	PEPLIST	DIFRIIMAI	DIFRIIMAI	0.0031	14.4191	0.0542	20.0842
0	QEKRHVLHL	PEPLIST	QEKRHVLH	QEKRHVLH	0.024	3.8028	0.0541	20.1428
0	SRRRQILHL	PEPLIST	SRRRQILH	SRRRQILH	0.0083	8.073	0.0522	21.1944
0	HGKRVVVAA	PEPLIST	HGKRVVV	HGKRVVV	0.0035	13.6709	0.0494	22.8427
0	KKRRSIVAV	PEPLIST	KKRRSIVA	KKRRSIVA	0.002	18.2525	0.0452	25.617
0	KKRRSIVAV	PEPLIST	KKRRSIVA	KKRRSIVA	0.002	18.2525	0.0452	25.617
0	AERRSVMAA	PEPLIST	AERRSVM	AERRSVM	0.0015	21.3901	0.0417	28.3027
0	AERRSVMAA	PEPLIST	AERRSVM	AERRSVM	0.0015	21.3901	0.0417	28.3027
0	VVKRFILHA	PEPLIST	VVKRFILH	VVKRFILH	0.0019	18.8485	0.0413	28.5849

0	VDYRGVLAC	PEPLIST	VDYRGVLA	VDYRGVLA	0.0015	21.0284	0.0388	30.9479
0	LDKRDIVHL	PEPLIST	LDKRDIVHI	LDKRDIVHI	0.0117	6.4078	0.0386	31.0808
0	YFKRVVLAA	PEPLIST	YFKRVVLA/	YFKRVVLA/	0.0012	23.9643	0.0374	32.3568
0	YFKRVVLAA	PEPLIST	YFKRVVLA/	YFKRVVLA/	0.0012	23.9643	0.0374	32.3568
0	NYCRHVLAA	PEPLIST	NYCRHVLA	NYCRHVLA	0.0005	34.8333	0.0367	33.1487
0	PKYRNVVAT	PEPLIST	PKYRNVVA	PKYRNVVA	0.0004	38.8148	0.034	35.9977
0	RICRMVLAT	PEPLIST	RICRMVLA	RICRMVLAT	0.0002	49.3333	0.033	37.2556
0	DFFRSVVAA	PEPLIST	DFFRSVVA.	DFFRSVVA.	0.0008	28.0606	0.0327	37.5464
0	GDKRAVLAM	PEPLIST	GDKRAVLA	GDKRAVLA	0.0027	15.7376	0.0319	38.5307
0	DVYRDILAC	PEPLIST	DVYRDILA(	DVYRDILA(	0.0007	29.2807	0.031	39.6491
0	GKKRSVMAF	PEPLIST	GKKRSVM/	GKKRSVM/	0.0025	16.3799	0.0299	41.1542
0	GKKRSVMAF	PEPLIST	GKKRSVM/	GKKRSVM/	0.0025	16.3799	0.0299	41.1542
0	RLWRVVLAC	PEPLIST	RLWRVVLA	RLWRVVLA	0.0005	33.975	0.028	43.9731
0	LLRRAVLAC	PEPLIST	LLRRAVLA(	LLRRAVLA(	0.0002	46.8462	0.0235	51.838
0	PVYRAVMAA	PEPLIST	PVYRAVM/	PVYRAVM/	0.0002	48	0.0221	54.6437
0	PKFRSIVHA	PEPLIST	PKFRSIVHA	PKFRSIVHA	0.0002	47.3846	0.0189	61.6397
0	PKFRSIVHA	PEPLIST	PKFRSIVHA	PKFRSIVHA	0.0002	47.3846	0.0189	61.6397
0	PKFRSIVHA	PEPLIST	PKFRSIVHA	PKFRSIVHA	0.0002	47.3846	0.0189	61.6397
0	PKFRSIVHA	PEPLIST	PKFRSIVHA	PKFRSIVHA	0.0002	47.3846	0.0189	61.6397
0	PKFRSIVHA	PEPLIST	PKFRSIVHA	PKFRSIVHA	0.0002	47.3846	0.0189	61.6397
0	QRRRAVVAC	PEPLIST	QRRRAVV/	QRRRAVV/	0.0002	51.4444	0.0159	69.0902
0	QRKRAVVAC	PEPLIST	QRKRAVV/	QRKRAVV/	0.0002	44.0625	0.0152	70.9584
0	QRKRAVVAC	PEPLIST	QRKRAVV/	QRKRAVV/	0.0002	44.0625	0.0152	70.9584
0	QRKRAVVAC	PEPLIST	QRKRAVV/	QRKRAVV/	0.0002	44.0625	0.0152	70.9584
0	PKRRDILAI	PEPLIST	PKRRDILAI	PKRRDILAI	0.0001	56.9697	0.0142	73.6035
0	PKRRDILAI	PEPLIST	PKRRDILAI	PKRRDILAI	0.0001	56.9697	0.0142	73.6035
0	DEKRAVLAT	PEPLIST	DEKRAVLA	DEKRAVLA	0.0001	55.1515	0.0107	83.8889
0	DIKRAVVHT	PEPLIST	DIKRAVVH	DIKRAVVH	0.0001	58.7879	0.0105	84.4062
0	EECRAVLHT	PEPLIST	EECRAVLH	EECRAVLH	0	71.1538	0.0105	84.4827
0	EECRAVLHT	PEPLIST	EECRAVLH	EECRAVLH	0	71.1538	0.0105	84.4827

Search Pattern: **x-x-[CRFKWY]-R-{KLPWYRTE}-[IV]-[VM**

Prosite, Uniprot/swissDb, Mus musculus

Ave	NB
0.4526	1
0.4526	1
0.4526	1
0.4526	1
0.2998	1
0.2652	1
0.2652	1
0.0435	0
0.0284	0
0.0319	0
0.0384	0
0.0093	0
0.0093	0
0.0244	0
0.0445	0
0.0445	0
0.0263	0
0.0404	0
0.0174	0
0.01	0
0.01	0
0.0078	0
0.0078	0
0.0028	0
0.0048	0
0.0048	0
0.012	0
0.0064	0
0.0142	0
0.0105	0
0.0083	0
0.0047	0
0.0065	0
0.0058	0
0.0058	0
0.0031	0
0.0031	0
0.024	0
0.0083	0
0.0035	0
0.002	0
0.002	0
0.0015	0
0.0015	0
0.0019	0

0.0015	0
0.0117	0
0.0012	0
0.0012	0
0.0005	0
0.0004	0
0.0002	0
0.0008	0
0.0027	0
0.0007	0
0.0025	0
0.0025	0
0.0005	0
0.0002	0
0.0002	0
0.0002	0
0.0002	0
0.0002	0
0.0002	0
0.0002	0
0.0002	0
0.0002	0
0.0002	0
0.0002	0
0.0001	0
0.0001	0
0.0001	0
0.0001	0
0	0
0	0

L]-[HA]-[ILMVCAFWT]
